# A Discussion of High-Risk HPV in a 6-Year-Old Female Survivor of Child Sexual Abuse

**DOI:** 10.1155/2017/6014026

**Published:** 2017-05-23

**Authors:** Connie D. Cao, Lena Merjanian, Joelle Pierre, Adrian Balica

**Affiliations:** ^1^Rutgers Robert Wood Johnson Medical School, Rutgers University, New Brunswick, NJ, USA; ^2^Department of Obstetrics, Gynecology, and Reproductive Sciences, Rutgers Robert Wood Johnson Medical School, Rutgers University, New Brunswick, NJ, USA; ^3^Department of Surgery, Rutgers Robert Wood Johnson Medical School, Rutgers University, New Brunswick, NJ, USA

## Abstract

**Background:**

Human papilloma viruses (HPVs) cause a variety of clinical manifestations in children including skin warts, laryngeal papillomas, and condyloma acuminatum. Whereas the mode of transmission is well understood and management of HPV infection is clearly defined by guidelines in adults, less is known about the mode of transmission, natural history of disease, and appropriate management of high-risk anogenital HPV infections in children.

**Case:**

The patient is a previously healthy 6-year-old female who presented with multiple vaginal lesions causing pain and discomfort and was diagnosed with HPV 18 positive CIN I.

**Summary and Conclusion:**

Children infected with high-risk HPV subtypes remain a vulnerable patient population, and there is minimal literature on the natural history of disease and effects of overtreatment. Based on a literature review, conservative management, HPV vaccination, and consideration of the cervical cancer screening guidelines for adolescent females are an appropriate treatment course until more studies are reported on cervical cancer screening in survivors of child sexual abuse.

## 1. Introduction

Human papilloma viruses (HPVs) are a family of small double-stranded DNA viruses, of which 30–40 genotypes infect the genital tract. The virus infects epithelial cells through abrasion of the skin or mucosa and can exist as a latent infection [1]. Low-risk types 6 and 11 are common causes of condyloma acuminatum. High-risk mucosal HPV types 16 and 18 have been implicated in the development of cervical, vulvar, penile, and pharyngeal malignancies. In children, there are several possible modes of transmission including perinatal vertical transmission, autoinoculation from nongenital cutaneous warts to the genitalia, and heteroinoculation from one individual to another; however, the observation of anogenital warts in children should trigger an evaluation for sexual abuse [1].

Little is known about the natural progression of high-risk HPV infection in children. It has been suggested that young children may be monitored for malignancy in a similar manner to adolescent females [1, 2]. Previous studies have demonstrated that adolescent and young women who test HPV positive with abnormal cervical cytologies can be managed with close follow-up and conservative management. Here, we discuss a pediatric patient who presented with vaginal lesions causing urinary discomfort following confirmed sexual abuse. The patient ultimately tested positive for HPV 18 and was diagnosed with CIN I.

## 2. Case Report

The patient is a premenarchal, 6-year-old female who presented to the emergency department (ED) with her mother because of mild discomfort with urination and “pimples” causing pain in the vaginal area. The patient was previously healthy and denied increased urinary frequency or urgency, vaginal bleeding, vaginal discharge, malaise, and fever. The patient and her mother denied inappropriate contact or touching. On physical examination, the patient was comfortable, smiling, and playful. The external pelvic examination was notable for multiple raised lesions on the labia minora and erythema of the external genitalia. No vesicles or vaginal discharge were observed. At the ED, a clinical diagnosis of genital warts was made.

Social Work and the Department of Child Protection and Permanency were contacted. The patient and her mother were referred to a state-designated child protection center that provides crisis intervention and child abuse assessments. At the state-designated child protection center, a team of trained professionals conducted an evaluation and was able to ascertain a history of abuse from the patient by an adult, male family member living in the household. The center conducted a thorough physical examination and infection screening.

Two months after initial presentation and evaluation at the child protection center, the patient's symptoms worsened secondary to an increase in the size of the lesion. The patient was able to urinate but unable to sleep at night secondary to pain and irritation caused by rubbing. On examination, the patient had warts in the vestibule that carpeted the space between the labia minora and fourchette, obliterated the vaginal introitus, and appeared to obscure the urethra.

**Figure 1:**
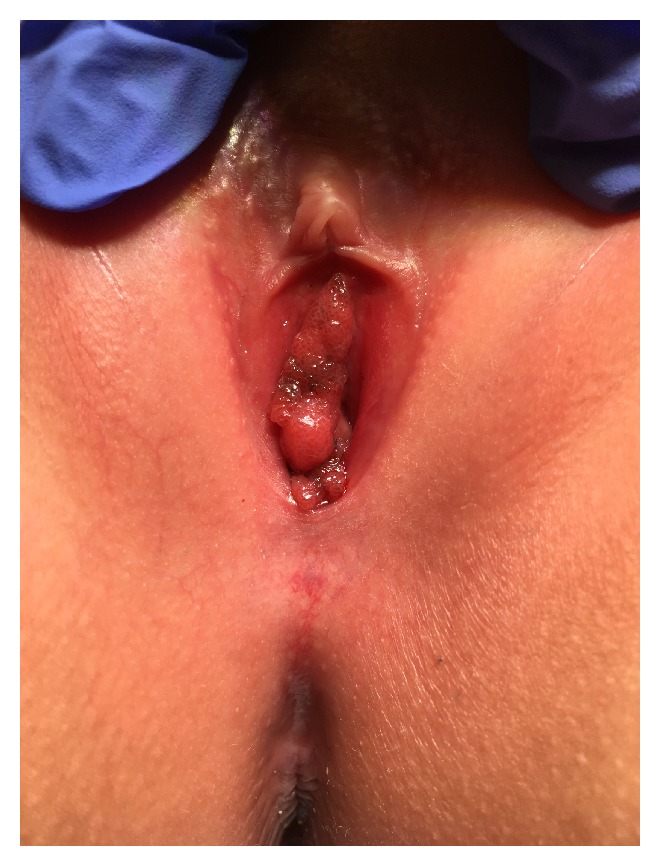
Photograph of the condylomas prior to excision.

**Figure 2:**
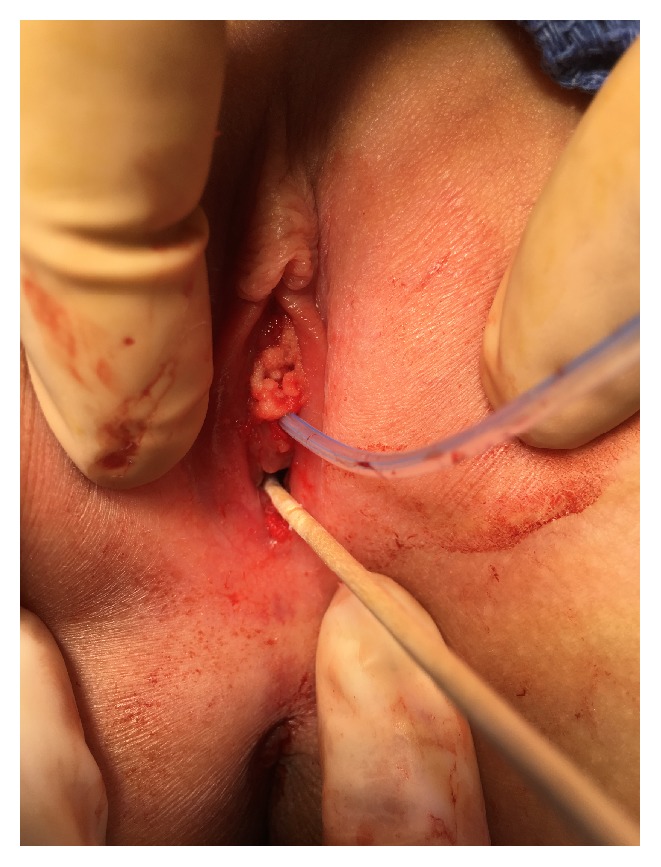
Photograph of the genital area after excision, with visualization of the urethra and vaginal introitus.

The patient then underwent an exam under anesthesia (EUA) and excision of vaginal lesions, as seen in Figure 1, in the lithotomy position by pediatric surgery and the gynecological team. Direct visualization of the area, without use of a hysteroscope, did not reveal any gross internal vaginal lesions. A sterile urinary catheter was placed. The condylomas were removed using smooth pickups and Metzenbaum scissors with careful visualization of the urethra, as in Figure 2. First, a 2 cm area of condyloma at the inferior portion of the vaginal introitus was removed. A second 2-3 cm lesion around the urethral meatus and third small lesion of the right labia minor were excised. Hemostasis was achieved with pressure and Surgiflo at the excision sites. A Vaginal ThinPrep Pap Test was performed and resulted in HPV 18 positive and low grade squamous intraepithelial lesion (LSIL) including cellular changes associated with HPV and cervical intraepithelial neoplasia I (CIN I). The surgical pathology consisted of red-tan, glistening, soft tissue that measured 1.2 × 1 × 0.4 cm when aggregated and was determined to be condyloma acuminatum. The patient was awakened from anesthesia and brought to the recovery room in stable condition.

At the postoperative follow-up visit, the mother and patient reported a smooth recovery with minimal pain. Exam of the surgical site in the supine frog-leg position showed that it was well healed with minimal residual disease. The patient was instructed to follow up with gynecology every 6 months and with pediatric surgery as needed. Given this patient falls outside CDC guidelines for vaccination and her pathology results, we plan to recommend HPV vaccination at the patient's next visit [3, 4].

## 3. Summary and Conclusion

The detection of a sexually transmitted infection (STI) in a child should raise suspicion for sexual abuse. Genital or anal condyloma acuminatum appearing for the first time in a child older than 5 years is likely to be the result of sexual transmission [5]. There is minimal safety and efficacy data on the medical treatment options for condyloma acuminatum in children younger than 12 years of age [6]. A retrospective chart-review study concluded that approximately half of the cases of anogenital warts in children spontaneously resolve [6]. As a result, nonintervention is a reasonable initial management approach for asymptomatic condylomas. Treatment options for symptomatic anogenital warts in children include local excision, laser-based ablation, cryotherapy, chemical ablation with trichloroacetic acid, podofilox gel, and imiquimod 5% cream [1]. For this patient, local excision was reasonable as the genital warts were large enough to cause symptoms. Since recurrence is possible with any treatment modality, long-term follow-up for children with anogenital warts is recommended.

While little is known about the epidemiology of HPV in children, one study demonstrated that 16% (5/31 girls) of child sex abuse victims are infected with HPV [2]. Factors associated with genital HPV infection as a result of child sexual abuse include female gender, genital warts, and increasing certainty of sexual abuse [7]. Currently, there have been no prospective studies conducted on a group of HPV-infected sexually abused children to assess the effects of age and immaturity of the reproductive tract on the course of HPV infection. As a result, researchers have proposed using studies of adolescents who voluntarily initiate sexual activity at the age of menarche as a guide to disease progression and appropriate management [2].

HPV infections behave differently in adolescent and young women when compared to older adult women [8]. Two explanations for the high rate of HPV infection in the adolescent population include the propensity for multiple sexual partners and physiology of the adolescent cervix. Hormonal changes during puberty cause cervical epithelial changes including metaplasia and the formation of a transformation zone, where squamous epithelial cells replace cervical columnar epithelial cells. Specifically, a rapid rate of squamous metaplasia was found to be a risk factor for the development of LSIL in HPV positive young women [9]. Additionally, less protective cervical mucous and larger size of the cervical ectropion during early adolescence increase the biological vulnerability of young women to genital HPV infection [10]. It has also been suggested that earlier sexual activity accelerates the process of cervical maturation [8].

The Papanicolaou (Pap) test is a screening tool for cervical cancer and has reduced the mortality rates of cervical cancer in the United States [11]. The Bethesda system standardizes reporting of cervical and vaginal cytology diagnoses by categorizing abnormal cytologies into four categories: atypical squamous cells (ASC), LSIL, high-grade squamous intraepithelial lesions (HSIL), and atypical glandular cells (AGC). With the Bethesda reporting in hand, clinicians apply the American Society of Colposcopy and Cervical Pathology algorithm for the management of abnormal Pap smears [12]. Current guidelines recommend beginning cervical cancer screening starting at the age of 21 every 3 years and HPV DNA cotesting starting at the age of 30 every 5 years [13].

These guidelines were based on studies of HPV in young women, which can provide insight regarding the management of this 6-year-old female since the natural progression of HPV in victims of child sexual abuse is not known. Although adolescent and young women have higher rates of HPV infection than older adult women, they are also more likely to clear the infection. One study noted that 65–75% of adolescent and young women were found to test negative for HPV DNA by 30 months. The same study suggested chronic HPV infection might be correlated with oncogenic, high-risk HPV viral types, indicating that high-risk viral types may more effectively evade the immune system [14]. Additionally, only 3% of young women with LSIL progressed to precancerous lesions [15]. Similarly, a different study indicated that 75% of the youngest adolescents (<16 years old) with CIN 2 had regressed within 2 years of follow-up [16]. Thus, conservative management is appropriate initial management in adolescent girls and young women with LSIL and commonly transient HPV infection [17].

Victims of child sex abuse are an especially vulnerable population for several reasons. Often, as in this case, the sexual abuse begins prior to the age at which HPV vaccinations are routinely given; the FDA has approved HPV vaccination starting at the age of 9 in females. This patient endured sexual abuse at a young age, placing a longer time period between the time of initial infection with HPV and onset of routine cervical cancer screening at the age of 21 [4]. Moreover, a consequence of child sex abuse often is increased high-risk behavior associated with cervical cancer such as sex work and substance use. Victims of sexual abuse are more likely to lose contact with health services and drop out from the education system than women who did not experience abuse. An Australian case-control study has identified unwanted sexual experiences involving genital contact as a potential prognostic factor for invasive cervical cancer by 25 years of age [3]. Even though the efficacy of the HPV vaccine decreases from 100% to 44% in those already exposed to HPV genotypes, it is imperative that the HPV vaccine is administered to victims of child sexual abuse soon after the abuse is reported [3, 4].

This case of a 6-year-old female victim of child sexual abuse infected with high-risk HPV highlights a vulnerable patient population where there is minimal understanding of the natural course of HPV infection and appropriate management. Further study is needed to ascertain the optimal management and appropriate time to perform a Pap smear [18]. In this case, the need to follow-up with the patient should be balanced with the anxiety and effect of psychosexual morbidity associated with increased cervical screening and colposcopy at such a young age [19]. Although young females are more likely to clear HPV infections than adults, a history of sexual abuse contributes additional risk factors for cervical cancer. Based on the review of the literature on HPV infection in adolescents and young women, we recommend close patient follow-up with gynecology and pediatric surgery for an exam of the external genitalia, with EUA and excision of condyloma as needed. We will consider starting cervical cancer screening at an earlier age of 18 years in such patients and HPV vaccination prior to the age of nine [3, 4].
